# Evaluating the centralized purchasing policy for the treatment of hepatitis C: The Colombian CASE

**DOI:** 10.1002/prp2.552

**Published:** 2019-12-10

**Authors:** Angela V. Pérez, Antonio J. Trujillo, Aurelio E. Mejia, Johnny D. Contreras, Joshua M. Sharfstein

**Affiliations:** ^1^ Johns Hopkins Bloomberg School of Public Health 101 North Wolfe Street Apt 415 Baltimore MD USA; ^2^ International Health Johns Hopkins Bloomberg School of Public Health Baltimore MD USA; ^3^ Health Technologies Ministry of Health and Social Protection Bogotá Colombia; ^4^ Ministry of Health and Social Protection Bogotá Colombia; ^5^ Johns Hopkins Bloomberg School of Public Health Baltimore MD USA

**Keywords:** Developing countries, Health policy, High‐cost drugs, Pharmacoepidemiology, Policy design, Pricing policy, Regulatory guideline, Strategic purchasing

## Abstract

The high cost of drugs for hepatitis C limits access and adherence to treatment. In 2017, the Colombian health care system decided to design a strategy. It consisted of centralized purchasing, regulations, clinical practice guidelines, and direct observation of the treatment and follow‐up of patients. The main objective of this study was to assess the centralized purchasing strategy in Colombia. The study design was a policy implementation assessment. We analyzed the change in prices, the clinical outcomes, and the opinions of stakeholders using data from the Ministry of Health. Additional information about effectiveness came from the Colombian Fund for High‐Cost Diseases and semi‐structured interviews of the stakeholders. The follow‐up was from October, 2017 to October, 2018. The total number of patients reported in the cohort period was 1069. The number that finished 12 weeks of treatment, completed the follow‐up for the case closure, and were considered cured through the end of October, 2018 was 563 (53%). The remainder, 506 patients (47%), are currently in treatment. A total of 543 of these treated patients (96%) were cured. After implementing this strategy, the drug prices decreased by more than 90% overall. Before implementation, the total direct cost was $100 102 171.75 dollars. Afterward, the cost was $8 378 747 dollars.

**Abbreviations**
CPGs: Clinical Practice Guidelines
DAA: Direct Acting Antiviral
WHO: World Health Organization

**Key point**

- High prices for hep C drugs and new technologies challenge the sustainability of health care systems.
- Designing policies that contribute to regulating drug prices is a priority for health care systems.
- The centralized negotiation and purchasing for hep C treatment will procure savings for the health care system.
- The centralized negotiation and purchasing for hep C treatment will lead to positive health results.

## 1 INTRODUCTION

The World Health Organization (WHO) estimates that there were 130 to 150 million cases of hepatitis C (hep C) infection worldwide in 2015, which caused approximately 1 300 000 deaths that year. This death rate is increasing over time.^1^ Hep C is a liver disease caused by a virus of the same name. The disease could be acute or chronic, with symptoms lasting from a few weeks to one's entire life.^2^

The major complications due to persistent viremia are liver fibrosis, cirrhosis, hepatocellular carcinoma, and death.^2^ These complications represent an essential burden of disability adjusted life years.

Innovation in the pharmaceutical industry is a critical factor in health care systems around the world. Some of the new drugs improve the health status and quality of life in communities. Hep C is not an exception. In the last few years, improvements have been made in the treatment of hep C, including Direct Acting Antiviral (DAA) drugs, which are better tolerated and have a higher effectiveness rate of 95% virological response than interferon‐based treatments.^3^

With access to medicine, the health burden caused by hep C could be lessened. Drugs to cure the disease are available. There are significant health benefits for patients, such as high rates of curation and high levels of drug safety. Likewise, for the health care system, the benefits include cost savings and a reduction in the population risk by decreasing transmission. Some of the spillover effects are: a positive impact on out of pocket expenditures and an improvement in the effective access to treatment. However, the price of these drugs is extremely high, which limits access.^4^

However, 97% of the Colombian population currently has the same coverage and guaranteed access to new technologies. The health care expenditure per person in Colombia is one‐fifth that of developed countries. Nevertheless, the inclusion of new technologies is crucial to maintaining the legitimacy of the system, but this situation represents a sustainability problem.^5^

Centralized purchasing has been identified as a possible solution. Colombia was the first country in the Americas to acquire this type of high‐cost treatment through the help of the Strategic Fund of the Pan American Health Organization (SF PAHO).^6^, ^7^

### 1.1 Centralized purchasing in Colombia

The National Viral Hepatitis Control Plan developed by the Colombian Ministry of Health and Protection (MSPS) had specific goals from 2014 to 2017. They were to “reduce the morbidity and mortality of viral hepatitis, cirrhosis, and hepatocellular carcinomas caused by chronic hepatitis infection by implementing strategies to promote health, disease prevention, timely diagnosis, and comprehensive care for infected patients.”^8^

As a result of implementing the national plan, the MSPS assessed two fundamental components to ensure compliance with the recommended actions. First, they sought to identify the availability of Clinical Practice Guidelines (CPGs), based on current research, for the treatment of viral hepatitis in the health care system. Second, they verified the prices in the national database, SISMED, and the availability of drugs for hep C in Colombia.

This evaluation identified the need to develop CPGs. As a result, the MSPS and the Health Technology Assessment Institute (IETS) created CPGs for screening, diagnosis, and treatment of patients infected by the hep C virus.^9^ At the same time, the cost and availability of new therapies for hep C was evaluated in Colombia.

In the reimbursement information system, it was also discovered that in 2016, the cost of a treatment course with Harvoni® was $108 546.15 dollars (340 032 758 Colombian pesos, based on an exchange rate of 3 132.61 Colombian pesos to the dollar). Even more shocking was the discovery that prices in Colombia for other molecules used to treat hep C, according to the reimbursement database, were higher than in other countries in the region. For example, the price of Sofosbuvir in Colombia was $72 586.16 dollars (227 384 136 Colombian pesos), while in countries such as Brazil and Argentina, the costs were $6786 and $6258 dollars, respectively. Daclatasvir had an average price of $15 209.61 dollars (47 645 778 Colombian pesos), while in Brazil and Argentina, the reported prices were $2550 and $3558 dollars, respectively.^10^

These findings were the motivation for designing a new strategy that would permit the reduction of drug prices and ensure access to treatment, an important part of the National Plan to Control Viral Hepatitis 2014‐2017. The MSPS explored various possibilities to purchase the drugs at lower prices, and they chose to adopt centralized drug purchasing through the SF PAHO.^11^

To carry out this plan, several players in the health care system needed to be involved including scientific organizations, patient advocate groups, the National Institute of Health (INS), the IETS, the Colombian Fund for High Cost Diseases (CAC), and different groups within the MSPS.^12^

A fundamental part of this strategy and work done by the relevant players was to develop Resolution 1692 in 2017 which, “establishes criterion for the centralized purchasing, distribution, and supply of drugs for chronic hepatitis C, along with follow‐up of patients with this diagnosis and other provisions”.^13^ The centralized purchasing took place in June, 2017, when the following drugs were acquired: Daclatasvir, Sofosbuvir, and Ledipasvir/Sofosbuvir.^7^

Given the relevance of the centralized drug purchasing strategy for hepatitis as a public policy in Colombia, the objective of this study was to evaluate its impact on the health care system, taking into account clinical, economic, and administrative impacts.

## 2 MATERIALS AND METHODS

The study design is a policy implementation assessment. The intervention was the centralized purchasing of treatments for hep C made by the Ministry of Health in Colombia. The endpoints were clinical effectiveness, savings for the health care system, and stakeholders’ perceptions. Information from the Ministry of Health was used, including the prices of the reimbursement for treatments, prices of the drugs purchased by means of this strategy, the quantity needed, and the number of cases with a confirmed diagnosis reported to the National Epidemiological Surveillance System. Additional information came from the CAC, which contained facets of clinical and pharmacological follow‐up. Also, they provided data on the effectiveness of the therapy, which is defined by the undetectable viral load 12 weeks after finishing the therapy, including all of the patients who received treatment as a result of the centralized purchase. Finally, other information came from semi‐structured interviews of the stakeholders.

### 2.1 Statistical analysis

We developed descriptive statistics of demographic information, summary measures, and measures of central tendency. We calculated and compared the differences in price and the savings before and after centralized purchasing. The quantity of drugs purchased was analyzed, with the assumption that a patient requires 84 doses for the complete treatment. The purchase prices used for the analysis were the prices reported in the purchase orders that the PAHO issued to the MSPS. The preliminary comparison price was the one reported by the MSPS through the reimbursement database from 2016. The currency exchange rate used was 3132.61 Colombian pesos to the dollar. With this information, the savings incurred by the centralized purchasing was estimated. Information provided by the CAC was used for the clinical evaluation of patients who received treatment as a result of this policy. The cohort of patients were those that began treatment after the policy took effect until October 31, 2018. For the evaluation of the effectiveness of the therapy, patients up through October 31, 2018 that had already finished treatment and had their viral load tested 12 weeks after treatment were included. Descriptive statistics on the population receiving treatment were obtained.

### 2.2 Qualitative analysis

In order to know and assess the opinions of the stakeholders, a qualitative approximation by way of semi‐structured interviews was used, which incorporated the following elements: (1) knowledge of the centralized purchasing strategy, (2) planning, (3) implementation, (4) results, (5) advantages of the strategy, (6) disadvantages, (7) opportunities for improvement, and (8) new technologies that would enable the use of similar strategies. Data analysis took place through thematic content analysis. For the interviews, responses were categorized into the strengths and weaknesses of the centralized purchasing strategy and suggested actions. From there, subcategories were created.

Stakeholders were selected by identifying who the key players in implementing this policy were.

## 3 RESULTS

### 3.1 Clinical results

Table 1 shows the characteristics of the population with the hep C diagnosis reported in the follow‐up cohort. The total number of patients to date that have undergone treatment for hep C through the strategy is 1069. The most frequent comorbidity reported was an infection from HIV, and the most prevalent genotype among the reported patients was 1B.

**Table 1:** Characteristics of the population that received treatment through the centralized purchase

Characteristics	n	%
Patients from the Cohort	1069	100
Female	522	49%
Male	547	51%
Average Age		
Female	64	
Male	50.4	
Department of Residence		
Bogotá	586	55%
Valle del Cauca	131	12%
Antioquia	99	9%
Atlántico	83	8%
Cundinamarca	52	5%
Other Departaments	118	11%
Comorbidity and Medical Background		
Hepatitis B	26	2%
HIV	248	23%
Chronic Kidney Disease ‐ Stages 4 and 5	35	3%
History of Cirrhosis	350	33%
History of Treatment for Hepatitis C	256	24%
Hepatitis C Genotypes		
Genotype 1	27	3%
Genotype 1 A	140	13%
Genotype 1 B	529	49%
Genotype 2	50	5%
Genotype 3	23	2%
Genotype 4	170	16%
Genotype 5	1	0%
Combination of Genotypes	6	1%
No Information	123	12%
Time of Diagnosis for Hepatitis C		
Less Than 1 y	159	15%
From 1 to 4 y	464	43%
From 5 to 9 y	137	13%
More than 10 y	164	15%
No Information	145	14%

Table 2 presents the status of patients from the cohort (n = 1069). 52% of these cases fulfill the necessary criterion defined under case closure (completion of the viral load 12 weeks after the final treatment). Of all the patients to date that meet the criterion for the case closure, cured patients and clinical effectiveness occurred in 69% of the cases. Only 4% could be considered therapeutic failures. Of the patients from the cohort who died during the follow‐up period, 8 had an autopsy report related to hep C. Of the 130 cases categorized as *other,* 7 patients (0.7%) left voluntarily and only 2 patients (0.2%) discontinued the treatment.

**Table 2:** Status of the patients from the cohort

Status	n	%
Patients with Case Closure	563	53%
Patients with Final Result – Cured	543	96%
Patients with Final Result – Not Cured – Failure	20	4%
Patients Currently in Treatment	155	14%
Patients that Finished Treatment, but did not Finish 12 weeks Post‐treatment	155	14%
Patients with a Viral Load Result Awaiting Post‐treatment	47	4%
Deaths	19	2%
Others	130	12%

In the Table S1, the types of treatment schemes for patients with case closure are shown. The majority of patients (96%) from the cohort with case closure received treatment with at least one drug from the centralized purchase (n = 456 patients). Of these, 96% (n = 440) were considered successes, meaning completely cured 12 weeks after the treatment.

### 3.2 Economic results

The centralized purchase of drugs for hep C included Daclatasvir, Sofosbuvir and Ledipasvir/Sofosbuvir. Table 3 shows the previous prices, the prices with the centralized purchase, the quantities purchased, and the savings obtained.

**Table 3:** Purchase prices and savings

Drug	Treatment Price^a^ Before the Centralized Purchase		Treatment Price with the Centralized Purchase	Quantity of Treatments Purchased	Total Cost Without Centralized Purchase ‐ US Dollars	Total Cost with Centralized Purchase ‐ US Dollars	Estimated Savings ‐ US Dollars
	Colombian Pesos	US Dollars					
Daclatasvir (60 mg)	$ 47 645 778.00	$ 15 209.61	$ 4 442.15	250	$ 3 802 402.63	$ 1 110 537.10	$ 4 448 285.25
Sofosbuvir (400 mg)	$ 227 384 136.00	$ 72 586.16	$ 5 626.47	250	$ 18 146 540.42	$ 1 406 618.51	$ 4 010 201.33
Sofosbuvir/Ledipasvir (400mg/90mg)	$ 340 032 758.00	$ 108 546.15	$ 8 141.10	720	$ 78 153 228.70	$ 5 861 591.52	$ 20 414 078.52
TOTAL					$ 100 102 171.75	$ 8 378 747.14	$ 91 723 424.61

a Treatment for 84 tablets

The cost of 250 treatments of Daclatasvir, 250 of Sofosbuvir and 720 of the combination Sofosbuvir/Daclatasvir with prices prior to the centralized purchase would have been $100 102 171.75 dollars. This same quantity, with the centralized purchase, had a cost of $8 378 747.14 dollars. The amount saved was $91 723 424.61dollars (287 333 717 181.77 Colombian pesos). This savings is an estimate based on the clinical consideration that the treatments would have a similar prescription pattern. This is supported by the fact that the drugs purchased were selected according to the evidence (safety and effectiveness) and based upon the professional opinions of clinical experts (hepatologists and infectologists). They considered this to be the prescription pattern.

### 3.3 Stakeholder opinions

Supporting information,Table S2 shows the stakeholders who participated in the semi‐structured interviews (see the online appendix).

*In the Supporting information, *Table S3 shows the results from the semi‐structured interviews of the key players in the health care system.

**Figure 1:**
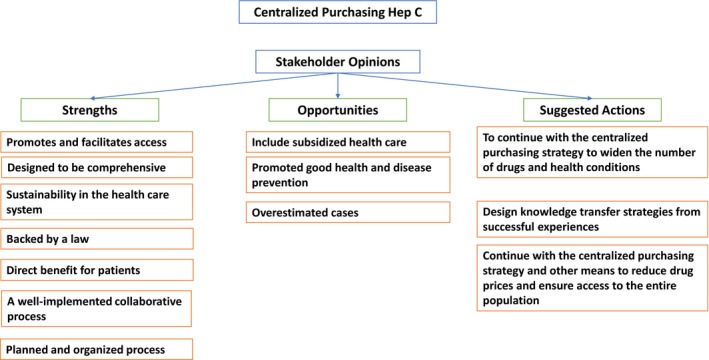
Summarized Stakeholder Feedback

Figure 1 shows the principal categories and most frequent comments made by stakeholders.

The responses were subdivided into strengths before and after implementation of the strategy. The interviewees felt that the strengths prior to implementation were related to the comprehensive strategy design. This is understood to mean that they include GPCs, pathways, regulations, and information systems. These are used for follow‐up, an adequate planning process that incorporates a review of information sources, prioritization of health conditions based on a review of the research, the participation of all of the players involved, and their commitment to the strategy.

Regarding strengths after the implementation, the interviewees felt that the strategy led to direct benefits for the health care system and the patients. This was due to the creation of cost‐effective actions, increased access to timely therapy, quality of care, and the fact that more than 98% of the patients adhered to the strategy. Of equal importance was the feeling that it was a strategy that involved an efficient use of resources and financial sustainability for the health care system. Finally, there was a direct benefit to the health care teams, evidenced by the positive impact on the health of the patients.

Several opportunities were identified regarding the centralized purchasing strategy. First, the calculation of quantities needed did not take into account the subsidized health care system. Also, there were some problems in primary care levels for screening and early detection. Moreover the prevalence of hep C was overestimated. The last issue was the lack of recognition of the strategy and its results by the academic world.

In terms of suggested actions for the future, the interviewees recommended continuing the centralized drug purchasing and negotiation strategy in a comprehensive way. In addition, they endorsed looking for new ways to identify and prioritize health conditions and technologies, strengthening training at primary care levels for screening, and striving for early detection for patients. Finally, they suggested including the subsidized health care system in these policies and strategies.

## 4 DISCUSSION

High drug prices are a burden on health care systems. This has been addressed by different price regulation policies including external reference pricing, annual price adjustments according to market competition, regulation of price increases, centralized purchasing and negotiation, regulation and evaluation of patent time frames, and regulation of price ceilings, among others.^14^, ^15^

The Colombian health care system has implemented a price regulation policy for drugs that includes three types of regulation: regulated freedom, direct control, and freedom under surveillance. This policy has been evaluated by several authors, including Prada et al, who have concluded that policies such as fixed pricing and direct control have reduced price inflation by almost 43%. However, the real pharmaceutical cost rose, mainly due to an increase in the quantities sold. As a result, the price fixing mechanism should be used together with market regulation.^16^

These findings are consistent with those reported by other authors, who consider that drug price regulation policies should be comprehensive and include more than one strategy, plus an analysis of micro‐ and macroeconomic factors.^14^, ^17^, ^18^, ^19^ In 2017, the Colombian health care system decided to include centralized drug purchasing for hep C as a strategy to reduce prices and increase access to treatment.

This is the first article to assess this policy for the treatment of hep C in Colombia. Implementation began one year ago, integrating several points of view: clinical results, economics, and the players in the health care system. Sources for official figures that indicate prices before and after the purchase were used. Also considered were the clinical results from the patient cohort and the opinions of key players through semi‐structured interviews.

Our findings suggest that the centralized purchasing strategy was successful for several reasons. Clinical effectiveness was found, evidenced by patients completely cured 12 weeks after treatment. Savings were obtained for the health care system due to a reduction in drug prices, a part of the negotiation.

The design process of the centralized purchasing strategy incorporated the analysis of epidemiological report statistics, the evaluation of research related to available treatments, future considerations, an analysis of reimbursements, price reduction mechanisms, and an analysis of the relevant players in the health care system. This enabled the creation of a comprehensive strategy that included several elements: the negotiation of drug prices by the SF PAHO; the monitoring of the delivery of drugs to the patients; the development of clinical practice guides, care pathways, operational and administrative pathways, information systems for clinical and logistical follow‐up, training and information processes, and a regulation that drove the strategy. It also incorporated the permanent interaction of all of the key players during the design and implementation process. All of these were critical factors for its success.

The most important elements of the plan that played an essential role in its success, aside from the centralized purchase, were the stakeholders’ participation and engagement with the strategy. The strategy was comprehensive and considered the perspectives of the government, insurance companies, patients, physicians, medical teams, pharmacists, and the pharmaceutical industry. This guaranteed transparency and successful implementation.

That said, there is still room for improvement. For example, the subsidized health care system was not included, so they were unable to access the same reduced prices for hep C treatment. However, it is worth mentioning that this did not limit the insurance companies from buying and delivering drugs to those who needed them within the subsidized system. One stakeholder comment was related to the calculation of the affected population, suggesting an overestimation of cases. This is a challenge for the information system and the strategies for centralized purchasing and negotiation, since one of the objectives was price reduction by aggregated demand. In addition, for the strategy to continue, it is critical to assure transparency between the key players, the health care system, and the pharmaceutical companies.^20^

One year after implementing centralized drug purchasing for hep C, all of the stakeholders agree upon the need to continue with the strategy and widen it to include other drugs and health conditions. At the same time, it is important to identify and assess other possible ways to lower prices and guarantee access and treatment quality for patients through comprehensive strategies that include the logistic chains, administrative activities, and clinical care.

The research only takes into account the financial savings due to lower drug prices. Future researchers should include reductions in medical care costs and economic productivity, as well, since this policy leads to a larger amount of individuals being cured with the same expenditure for the Colombian health care system.

It is important to mention that we are talking about one‐year savings, since companies often raise prices rapidly after the first two years. Future work should evaluate the long‐term consequences of the policy.^21^, ^22^ On another note, companies may adopt strategic behavior and bundle prices for different drugs. This implies lower costs for hep C drugs and higher prices for other drugs not included in the centralized purchase.

This research has some limitations, one of which is not including the analysis of drug prices for the subsidized health care system because it was not included in the centralized purchasing. Also, there is a lack of clinical results from this population, given that the information system designed to record them did not include the report from the subsidized system.

Health care systems around the world have implemented different strategies to reduce drug prices and improve access. The main objective is to look for alternative options for the future. One choice is the Netflix Model, in which a subscription fee is paid to a pharmaceutical company. This would reduce the possibility of strategic behavior from these companies, ensure that patients have access to drugs, and decrease inequalities.

## 5 CONCLUSION

The centralized drug purchasing strategy for hep C developed and implemented by the Colombian Health Ministry was a success. The creation of policies designed to reduce drug prices and improve access and quality for patients to achieve improved health results should be a priority of the health care system. It should allow these implemented strategies to continue, along with a comprehensive plan for innovations in new strategies.

## DISCLOSURES

None of the authors have a conflict of interest.

## AUTHORS' CONTRIBUTIONS

Angela V. Pérez and Johnny D. Contreras authors are Designed research, conducted research, analyzed data, wrote paper, responsibility for the final content. Antonio J. Trujillo, Aurelio E. Mejia and Joshua M. Sharfstein authors are Analyzed data, wrote paper, responsibility for the final content.

## ETHICAL APPROVAL/STATEMENT EA NOT REQUIRED

This research does not require an ethical approval because this study analyzed secondary data, the information is public, and our analysis is aggregated.

The interviewers approved the publication of the results of the interview because the data and analysis were aggregated.

## STATEMENT ABOUT PRIOR POSTINGS AND PRESENTATIONS

The abstract of this research was accepted for a poster presentation at the 35th International Conference on Pharmacoepidemiology and Therapeutic Risk Management (ICPE 2019).
