# Supplementary material for: Evaluating the centralized purchasing policy for the treatment of hepatitis C: The Colombian CASE
Source: Pharmacol Res Perspect. 2019 Dec 10;7(6):e00552. doi: 10.1002/prp2.552 (PMC6902741; doi:10.1002/prp2.552)
Supplement: Supplementary file 1 [file PRP2-7-e00552-s001.docx]

**Online Appendix**

***Supporting information* *Table 1* Therapeutic Schemes and Case Closure Status**

| **Therapeutic Scheme** | **Cured** | **Number of Patients** | **% Cured** |
| --- | --- | --- | --- |
| Ledipasvir - Sofosbuvir | 243 | 257 | 95% |
| Daclatasvir - Sofosbuvir | 124 | 124 | 100% |
| Viekira | 92 | 96 | 96% |
| Daclatasvir - Sofosbuvir - Ribavirina | 25 | 26 | 96% |
| Daclatasvir - Asunaprevir | 21 | 21 | 100% |
| Ledipasvir - Sofosbuvir - Ribavirina | 14 | 15 | 93% |
| Simeprevir - Sofosbuvir | 10 | 10 | 100% |
| Viekira - Ribavirina | 4 | 4 | 100% |
| Interferón - Ribavirina | 2 | 2 | 100% |
| Simeprevir - Sofosbuvir - Ribavirina | 2 | 2 | 100% |
| Interferón - Simeprevir - Ribavirina | 1 | 1 | 100% |
| Viekira - Sofosbuvir | 1 | 1 | 100% |
| Viekira - Dasabuvir | 1 | 1 | 100% |
| Daclatasvir - Sofosbuvir - Ribavirina | 1 | 1 | 100% |
| Sofosbuvir - Elbasvir/ Grazoprevir - Ribavirina | 1 | 1 | 100% |
| Simeprevir - PEG - Ribavirina | 1 | 1 | 100% |

***Supporting information Table 2*** **Stakeholders Who Participated in the Semi-Structured Interviews**

| MSPS – Department of Drugs and Health Technologies, the Health and Social Protection Ministry – Department of Health Promotion and Prevention |
| --- |
| The Colombian Association of Business in Comprehensive Medicine (ACEMI) |
| Colombian Fund for High Cost Diseases (CAC) |
| Gestarsalud |
| Resource Manager of the General Social Security System for Health |
| Insurance Company in the Contributive Health Care System |
| Health Services Company |
| National Association of Colombian Businessmen (ANDI) – Health Section |

***Supporting information Table 3*** **Strengths, Opportunities, and Suggested Actions**

| **Strengths** | **Opportunities** | **Suggested Actions** |
| --- | --- | --- |
| The centralized purchasing strategy is innovative and **promotes and facilitates access** to new technologies with a better profile for safety and drug effectiveness. This strategy also fosters **sustainability in the health care system** through significant savings for contributory health care. Another key aspect of this strategy was the **effectiveness of the therapy**, which, according to the report from the Colombian Fund for High Cost Diseases, was 95%. A fundamental element that led to the success of this strategy was the fact that it was **designed to be comprehensive**, since it included the drugs as well as clinical and logistical follow-up. This led to an efficient use of resources. | This strategy only included the contributory health care. A change is currently being made to the law to **include subsidized health care.** | The great challenge is the logistics of delivering the drugs and the supervision of the patients in taking them. One goal is to **guarantee that the patients adhere to the treatment. Another goal is to continue with the centralized purchasing strategy to widen the number of drugs and health conditions**. In addition, it is important to continue to identify mechanisms that lead to an efficient use of resources, improving access to drugs and achieving optimal health results. |
| The centralized drug purchasing strategy was a **planned and organized process** that capitalized on many **sources of information** within the health care system. **Different players in the health care system participated** (scientific organizations, patient advocacy groups, ministry management, the IETS, the CAC, and the pharmaceutical industry, among others), enabling a successful implementation.  **The information and follow-up systems designed** for this strategy were key components, since they enabled the purchasers to maintain control of the drugs purchased and patient follow-up, leading to positive economic and health results. A critical factor was that the strategy was **backed by a law** (Resolution 1692 from 2017). | **Including subsidized and other health care systems was important, as well as the active search for patients** and the availability of resources for **actions that promoted good health and disease prevention.** | **Keeping this strategy going**, incorporating new drugs for the treatment of hep C, and updating the GPCs and clinical pathways are all areas to consider. |
| Not only did relevant players **participate in designing** the strategy and identifying the problem, but they also designed the solution. As a result, there was widespread acceptance and a commitment from the insurance companies, clinics, and hospitals involved. The result was an effective implementation. | Despite this being a successful strategy, there was **no recognition given from the academic world**. There are no human resources training in health oriented towards **primary care that could limit the processes of screening and early detection.** | **Designing knowledge transfer strategies from successful experiences** will help cause a paradigm shift. We must **continue working on strategies and models to reduce drug costs** and find different delivery and distribution methods. |
| The calculation to determine the quantity of drugs needed was a **collaborative process that was well-implemented.** | **Subsidized health care was not part** of the centralized purchase, due to the fact that the population that uses it is more vulnerable. | **Continuing with the centralized purchase strategy and other means to reduce drug prices and ensure access to the entire population are important components. Subsidized health care** would benefit from these policies, as well. Documentation of the expenses for those outside of the mandatory drug plan within subsidized health care would be useful. |
| The centralized purchase led to benefits to the health care system in the following areas: 1) e**conomics and sustainability**, 2) reduction of overcharging by middlemen, 3) a process free from corruption, and 4) **direct benefits for patients.** | **Overestimated cases** generated false hope for the pharmaceutical industry about the quantities involved. The reason for choosing hep C for this process was not clear, considering that there are many other health conditions that are higher priorities. | **Continuing the centralized purchase policy, looking for other cost reduction strategies, and increasing access to drugs** could all improve the situation. Transparency in the prioritization and negotiation processes would be a plus. |
| **Clear priorities** were established that explained the reasons why **hep C** was considered as a basis for this strategy (prices, prevalence, high financial impact). | **Not all of the available treatments nor the mandatory health plan were part of the negotiation, given the fact that the pharmaceutical laboratories involved with SF PAHO are free to accept the conditions of this fund or not.** For this reason, one particular laboratory chose not be a part of the centralized purchase. | **Continuing with the negotiation and centralized purchase processes,** assessing the possibility of incorporating other organizations aside from SF PAHO, and **including subsidized health care in these processes** would be **useful**. |
| This was a **cost-effective** strategy. Prior to implementing the centralized purchasing strategy, there was no clear, standardized clinical follow-up. Access to the drugs was inadequate and the risk of failure and resistance was high. Now, with the centralized purchasing strategy, **clinical follow-up is clear, drug delivery is on time**, and we know the positive health results. For the insurance companies, the costs dropped significantly and there is active patient management. A critical factor for success was **designing a complete strategy that included GPCs, pathways and a regulation**. | Some hospitals lack the capacity to implement this strategy, which limited the insurance companies in providing care to some patients that do not reside in urban centers. Despite this, they managed to overcome these challenges. | **Continuing with these kinds of strategies and looking for similar strategies to include other health conditions would be beneficial.** The Health Ministry should continue in this coordinating role in order to implement policies that involve participation by the key players. |
| Before this strategy, the institutions that provided health care services reported to the epidemiological surveillance system less frequently. They didn’t know the clinical result of the patients. After implementing the strategy, reporting to the surveillance system increased, as it is part of the strategy. The clinical status of the patients is known, and the **cured patients have motivated everyone on the health care team**. The acceptance level of the **supervised delivery by the patients was above 98%, an excellent level**. | **A low level of early detection and screening** caused some patients to seek treatment at advanced stages of the disease. | **Continuing with the centralized purchasing strategy, strengthening training for primary health care** for screening, and incorporating new technologies and new health conditions in similar strategies would all be relevant. |
| For the insurance companies, it was an optimal strategy. The high cost burden is gone, the **budget is clear, and the negotiation is fair**. The health care budget and management of the high cost drug expense are responsibilities of the government. A successful strategy was designed to handle these. From the government’s point of view, the goals were achieved. The advantage for the pharmaceutical industry is that now the government makes centralized purchases. As a result, the companies can avoid direct payment to the insurance companies, which brings benefits to the health care system. This strategy allows them to avoid the creation of a black market and can lead to **patient awareness that the strategy was designed for their wellbeing.** | **The calculation of the quantities needed was not accurate.** | A paradigm shift related to the contracting process by these entities is needed. Changes involving the treatment phase and **intervention in early diagnosis and risk adjustment** are necessary, since we are talking about a model oriented towards treatment and not prevention. Another important idea is to **continue with similar strategies that would allow other health conditions to be included to maximize the benefits for patients.** |
